# Anti-Cancer Strategy Based on Changes in the Role of Autophagy Depending on the Survival Environment and Tumorigenesis Stages

**DOI:** 10.3390/molecules29215134

**Published:** 2024-10-30

**Authors:** Michael Lee, Hye-Gyo Kim

**Affiliations:** 1 Division of Life Sciences, College of Life Sciences and Bioengineering, Incheon National University, Incheon 22012, Republic of Korea; 2 Institute for New Drug Development, Incheon National University, Incheon 22012, Republic of Korea

**Keywords:** canonical autophagy, alternative autophagy, cellular transformation, tumor, microenvironment, anticancer drugs

## Abstract

Autophagy is a crucial mechanism for recycling intracellular materials, and under normal metabolic conditions, it is maintained at low levels in cells. However, when nutrients are deficient or under hypoxic conditions, the level of autophagy significantly increases. Particularly in cancer cells, which grow more rapidly than normal cells and tend to grow in a three-dimensional manner, cells inside the cell mass often face limited oxygen supply, leading to inherently higher levels of autophagy. Therefore, the initial development of anticancer drugs targeting autophagy was based on a strategy to suppress these high levels of autophagy. However, anticancer drugs that inhibit autophagy have not shown promising results in clinical trials, as it has been revealed that autophagy does not always play a role that favors cancer cell survival. Hence, this review aims to suggest anticancer strategies based on the changes in the role of autophagy according to survival conditions and tumorigenesis stage.

## 1. Introduction

Autophagy is a cellular process in which intracellular materials are delivered to the lysosome for degradation [1], and refers to the concept that cells can survive through the recycling of materials under nutrient-deprived conditions. Yoshinori Ohsumi, awarded the Nobel Prize in Physiology or Medicine in 2016 for his discovery of autophagy mechanisms, identified several autophagy-related genes that are known as APG through yeast genetic screening [2]. Since autophagy plays a crucial role in degrading unnecessary proteins and cellular organelles, its dysregulation is associated with several diseases, including neurodegenerative disorders [3,4,5], as well as malignant tumors such as those of the liver, colon, stomach, and breast [6]. In particular, there has been significant research on the role of autophagy in tumorigenesis, with many studies reporting the development of liver tumors in autophagy-deficient mouse models [7,8,9]. Our laboratory has also confirmed that the loss of *ATG5* induces cellular transformation [10].

It has been reported that a type of autophagy called alternative autophagy exists, which shares similarities with canonical autophagy but operates independently of ATG5/ATG7 [11,12]. Although the exact role and mechanism of alternative autophagy remain unclear, there is a key difference in the membrane source. Canonical autophagy originates from the endoplasmic reticulum (ER) and mitochondria-associated ER membranes, whereas alternative autophagy originates from the trans-Golgi membrane [11]. In addition, in canonical autophagy, ATG5, ATG7, and LC3 are involved in autophagosome formation. However, in alternative autophagy, the Rab9 protein, which participates in late endosome-trans-Golgi transport, attaches to the autophagic membrane, leading to the generation of autophagosomes and autolysosomes [13,14]. Studies have shown that alternative autophagy plays an important role in the pathophysiological processes of various diseases, including cardiac diseases [15], neurodegenerative diseases [14], and tumorigenesis [16,17,18]. Notably, alternative autophagy has been reported to be primarily involved in mitochondrial clearance [19]. Alternative autophagy appears to be induced only under cellular stress conditions rather than under basal conditions. Our laboratory has reported that the higher viability of Ras-transformed cells compared to that of normal cell lines during long-term culture is due to relatively higher levels of alternative autophagy [20,21]. Furthermore, when treated with paclitaxel, a natural anticancer drug commonly used to treat several types of cancer, Ras-transformed cells showed a significant reduction in alternative autophagy and increased cell death, suggesting that alternative autophagy plays a crucial role in the survival of Ras-transformed cells [21].

Increasing evidence has linked autophagy closely to drug resistance and immune evasion in tumor cells [22]. Canonical autophagy has been demonstrated to function at each maturation stage of dendritic cells, including antigen presentation and cytokine secretion [23]. Additionally, alternative autophagy has also been shown to take over the role of canonical autophagy in regulating bacterial phagocytosis, as well as cell differentiation and maturation [24].

**Figure 1:**
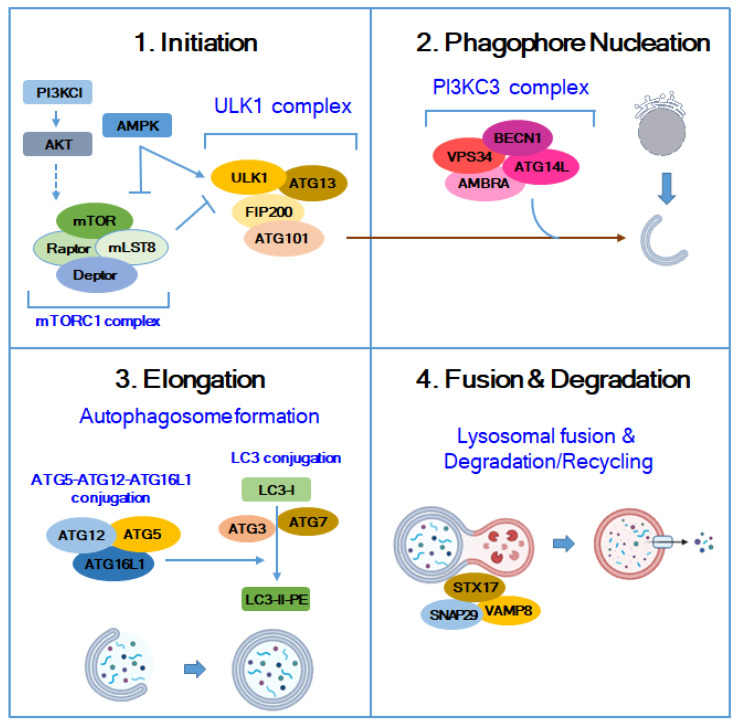
The four stages of autophagy. Autophagy is a process divided into four main stages: initiation, phagophore nucleation, elongation, and fusion/degradation. During the initiation stage, signaling proteins respond to external factors such as stress stimuli to regulate autophagy. When cellular energy levels are low, AMPK directly activates ULK1 to induce autophagy, while the type I PI3K/AKT pathway inhibits it via mTOR. In the nucleation stage, BECN1 serves as a platform, forming core complexes with proteins like VPS34 (a type III PI3K), AMBRA, and ATG14L. The elongation stage involves two ubiquitin-like conjugation systems: LC3 and ATG12. ATG12 forms a complex with ATG5, playing a crucial regulatory role in early autophagosome formation and interacting with ATG16L to create larger multiprotein complexes. LC3 is activated by ATG7 and transferred to ATG3, where it binds to the lipid PE through the ATG12–ATG5 conjugate, producing LC3-II, a key marker of autophagy. Once the autophagosome is fully formed, it fuses with lysosomes to create autolysosomes, a process requiring SNARE proteins such as STX17, SNAP29, and VAMP8. In the autolysosome’s acidic environment, the inner membrane is degraded, and the contents are broken down by hydrolytic enzymes.

Autophagy is divided into four major steps: initiation, nucleation, elongation, and fusion/degradation (Figure 1). Any protein involved in these four steps could be a target for anticancer drugs. In the initiation step, signaling proteins respond to external factors such as stress stimuli. When the cellular energy levels are low, AMPK directly activates ULK1 to induce autophagy, whereas the type I PI3K/AKT pathway inhibits autophagy via the mTOR pathway. In the nucleation step, BECN1 acts as a platform to form a core complex with proteins, such as VPS34 (type III PI3K), AMBRA, and ATG14L. Two ubiquitin-like conjugation systems are involved in the elongation step. ATG12 plays an important regulatory role in initial autophagosome formation by forming a complex with ATG5 and interacting with ATG16L to form a larger multiprotein complex. LC3 binds to the lipid PE through the ATG12–ATG5 conjugate to generate LC3-II, a key marker of autophagy. Once the autophagosome is fully formed, it fuses with lysosomes to create autolysosomes, a process that requires SNARE proteins, such as STX17, SNAP29, and VAMP8. To date, several autophagy regulators have been approved by the U.S. Food and Drug Administration (FDA) for cancer treatment and many others are currently under research and development [25,26,27,28,29,30].

## 2. The Role of Autophagy in Different Stages of Cellular Transformation

As the impact of autophagy on tumor formation and development has been increasingly recognized, strategies targeting autophagy have been continuously explored as efficient approaches for cancer therapy. Since autophagy is a mechanism that continuously degrades proteins and organelles to maintain normal function and protect against adverse stress, it was initially considered that autophagy inhibition might be effective in treating cancer [31]. However, it has been revealed that depending on the type and developmental stage of the tumor, autophagy activation or inhibition can contribute differently to tumor formation [31,32,33,34,35]. In particular, the role of autophagy varies according to the stage of tumorigenesis [36]. In normal cells, autophagy has a tumor-suppressive effect, and its absence may exacerbate the early stages of tumor development [10]. In RAS-induced cancers, autophagy blocks further progression to malignancy [37]. According to Sun et al. [38], autophagy suppresses liver cancer formation during the dysplastic stage but promotes it during the tumor-forming stage.

### 2.1. The Tumor-Suppressive Role of Autophagy

Many reports have indicated that autophagy prevents cancer development in the early stages of tumor formation [31,32,39,40]. Autophagy is a highly adaptive process for maintaining cellular homeostasis and preventing tumorigenesis in response to various forms of cellular stress [41,42,43,44]. Mitochondrial damage leads to the accumulation of reactive oxygen species (ROS), which can cause DNA damage and promote tumorigenesis [45,46,47,48]. Autophagy helps prevent ROS accumulation, which can cause DNA damage associated with cancer progression by removing damaged mitochondria [6,49,50]. Additionally, Beclin-1, an essential mediator of autophagy, acts as a tumor suppressor, protecting cells from unfavorable stimuli and stress in early cancer stages [51,52]. In fact, in various tumors, such as breast and prostate cancers, monoallelic deletion of Beclin-1 and inactivation of autophagy have been observed [53,54,55,56]. Furthermore, decreased expression of autophagy-related genes such as *ATG5*, *ATG7*, and *Beclin-1* has been observed in hepatocellular carcinoma (HCC) cells [57], and our laboratory has confirmed that autophagy deficiency due to *ATG5* knockout can induce malignant cellular transformation [10].

### 2.2. The Tumor-Promoting Role of Autophagy

Autophagy is a survival mechanism that occurs in response to stress. In established tumors, autophagy provides an alternative route to supply essential nutrients, supporting the increased metabolic activity associated with explosive cell growth and thereby meeting the energy demands of cancer cells [58,59,60]. Autophagy has also been shown to promote immune evasion in tumor cells [61,62]. Furthermore, autophagy upregulation may induce resistance to cancer therapy [26]. Using a mouse model of cancer induced by oncogenic Ras, Ying et al. [63] demonstrated that autophagy is required for tumor development. In our laboratory, we have shown that Ras-transformed cells maintain higher levels of basal autophagy than parental cells [20,64].

## 3. Changes in Autophagy Levels Based on the Survival Environment of Transformed Cells

When the tumor microenvironment is subjected to stress conditions such as starvation, hypoxia, and ROS accumulation, tumor cells induce autophagy to maintain metabolic homeostasis [65,66]. In highly proliferative cancer cells, the increased metabolic demands and poor vascularization of solid tumors often lead to hypoxic conditions in the tumor microenvironment. As a result, adaptive metabolic responses are promoted because cells cannot meet the high demands for amino acids, oxygen, and growth factors [67,68,69]. The relationship between hypoxia and autophagy as a survival mechanism has been demonstrated in various cancer and normal cells [70]. However, our recent laboratory studies have shown that increased cell density due to long-term culture results in a decrease in canonical autophagy and a relatively greater role of alternative autophagy in transformed cells [20]. Furthermore, the importance of mitochondrial clearance in tumor suppression is evidenced by the accumulation of damaged mitochondria and the subsequent accumulation of ROS and DNA damage in cells with the deletion of key autophagy genes [71]. Our laboratory observed that Ras-transformed cell lines generated higher ROS levels than normal cell lines [21]. In particular, under unfavorable culture conditions due to long-term culture, parental NIH 3T3 cells continuously reduced their mitochondrial mass, whereas Ras-transformed NIH 3T3 cells maintained a high mitochondrial mass. Recent studies confirmed the functional significance of alternative autophagy in the removal of damaged mitochondria [19]. Therefore, while tumor cells generally rely on increased canonical autophagy to supply nutrients, such as metabolites, the role of alternative autophagy in removing damaged mitochondria may become more critical in unfavorable survival conditions.

## 4. The Relationship Between Alternative Autophagy and Cancer

Nishida et al. [11] reported the existence of Rab9 protein-dependent alternative autophagy, which is involved in transport between endosomes and the trans-Golgi network, even in the absence of major ATG factors during etoposide-induced autophagy. Subsequently, it was found that almost all cells possess both canonical and alternative autophagy mechanisms [72] and that different types of autophagy can be induced depending on the type of substrate or cellular stress. For instance, starvation primarily induces canonical autophagy, whereas genotoxic stress activates both types of autophagy [11]. Alternative autophagy has been reported to be associated with age-related diseases such as aging and neurodegenerative disorders; however, it has also been noted that tumor cells have adapted to utilize non-canonical alternative autophagy pathways to support tumor growth and progression [73]. To meet the increased metabolic demand of established tumor cells, inhibition of hyperactivated autophagy tends to promote alternative autophagy [74]. Recently, our laboratory showed that Ras-transformed cells exhibited reduced canonical autophagy upon long-term culture, resulting in increased resistance to the autophagy inhibitor chloroquine [20]. This suggests that as tumor cell confluency increases, canonical autophagy may no longer be essential for tumor cell survival. Furthermore, in Ras-transformed cells, increasing cell density enhances alternative autophagy and increases resistance to the chemotherapeutic drug paclitaxel [21,75]. In addition, inhibition of alternative autophagy with Brefeldin A or knockdown of Rab9 significantly reduced the viability of Ras-NIH 3T3 cells. These results suggest that alternative autophagy is more important than canonical autophagy in maintaining cell survival under adverse conditions such as high cell density and anticancer drug exposure [21]. Therefore, the physiological relevance and molecular mechanisms mediating alternative autophagy in cancer may be attractive therapeutic targets, alongside canonical autophagy.

In fact, substances specifically regulating alternative autophagy have not yet been reported. However, anticancer drugs such as etoposide [76], camptothecin [77], and staurosporine [78] are known to activate alternative autophagy. Meanwhile, canonical autophagy modulators such as bafilomycin A1 [11], 3-methyladenine [11], wortmannin [78], and chloroquine [79] have also been reported to inhibit alternative autophagy. Brefeldin A [11], a widely used selective inhibitor of alternative autophagy, is known to increase the cytotoxicity of imatinib, a targeted cancer drug for chronic myeloid leukemia [80]; however, the relationship between this mechanism of action and the inhibition of alternative autophagy has not been reported. Consequently, to elucidate the role of alternative autophagy in tumorigenesis, it is necessary to develop new autophagy modulators that selectively target the autophagy signaling molecules essential for the alternative autophagy pathway.

## 5. Current Status of Anticancer Drug Development Using Autophagy Regulators

Autophagy regulation has emerged as a promising approach for cancer treatment and has become a significant target in the field. However, the dual role of autophagy in tumor growth and development poses a major challenge for the development of anticancer drugs that target autophagy. Therefore, anticancer drugs targeting autophagy should be differentiated using inhibitors or inducers depending on the stage of tumor development (Table 1). In the excessive growth stage, cancer cells rely on autophagy to survive under unfavorable conditions, such as nutrient deprivation and hypoxia, making it an attractive therapeutic target because of its high energy requirements. Consequently, many anticancer drugs target autophagy through inhibitory strategies. Moreover, the inhibition of autophagy renders cancer cells more susceptible to the cytotoxic effects of conventional anticancer drugs, thereby improving therapeutic outcomes in a variety of cancers, including breast and ovarian cancers and melanoma [81,82,83,84]. However, given the developmental nature of tumors, they are often discovered in patients only after they have progressed beyond their initial developmental stages. Considering the dual nature of autophagy, some studies have focused on activating autophagy along with other conventional treatments to directly induce cell death. Uncontrolled and continuous activation of autophagy can ultimately lead to cell death [85]. Excessive autophagy is sometimes induced via pharmacological or genetic treatments to prompt tumor cell death. However, owing to the complexity of autophagic physiology, there is still an intense debate about whether autophagy should be activated or inhibited, and no consensus has been reached.

**Figure 2:**
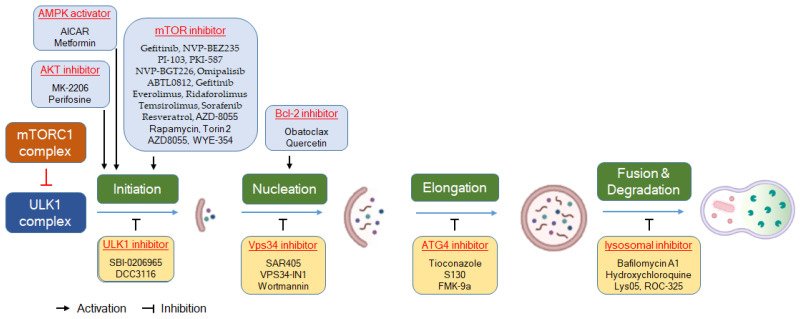
Autophagy modulators targeting each step of the autophagy pathway.

As shown in Table 1, anticancer drugs developed to induce autophagy often target proteins that regulate the initiation stages, particularly mTOR. On the other hand, anticancer drugs developed as autophagy inhibitors target almost every stage of autophagy, from vesicle nucleation and maturation to vesicle fusion and lysosomal degradation. In the development of autophagy inhibitors, the target protein at the initiation stage is primarily ULK1. These inhibitors function by forming complexes with the ULK1 regulatory unit and interfere with kinases in clinical settings, making ULK1 an attractive candidate for autophagy inhibition [86]. Inhibitors targeting type III PI3K (VPS34), at the nucleation stage, which is involved in autophagy and vesicle dynamics, have been developed [87,88,89]. Inhibitors that target the maturation stage mainly include autophagosome–lysosome fusion inhibitors, which inhibit lysosomal acidification and consequently disrupt autophagy [90,91]. Additionally, the potential of proteasome inhibitors to suppress autophagy has been explored as a promising therapeutic strategy for cancer [92]. Figure 2 also shows autophagy modulators targeting each step of the autophagy pathway.

**Table 1:** Pharmacological inducers and inhibitors targeting autophagy for anticancer therapy.

Autophagy Stage	Target	Drug	Target Tumor	Refs.
		**Inducer**		
Initiation	Akt inhibitor	MK-2206	Glioma	[93]
	Akt inhibitor	Perifosine	Colorectal cancer	[94]
	AMPK activator	AICAR	Renal cancer	[95]
	AMPK activator	Metformin	Hepatocellular carcinoma, colon cancer	[96,97]
	ULK1 activator	LYN-1604	Breast cancer	[98]
	PI3K/Akt/mTOR	Gefitinib	Lung cancer	[99]
	dual class I PI3K/mTOR inhibitor	NVP-BEZ235	Multiple myeloma	[100]
	PI3K/mTOR inhibitor	PI-103	Acute myelogenous leukemia	[101]
	PI3K/mTOR inhibitor	PKI-587	Hepatocellular carcinoma	[102]
	PI3K/mTOR inhibitor	NVP-BGT226	Hepatocarcinoma	[103]
	PI3K/mTOR inhibitor	Omipalisib (GSK2126458)	Esophageal squamous cell carcinoma	[104]
	Akt/mTOR inhibitor	Salvianolic acid B	Colorectal cancer	[105]
	Akt/mTOR inhibitor	ABTL0812	Advanced solid tumor	[106]
	Akt/mTOR inhibitor	Gefitinib	Non-small cell lung cancer	[99]
	PAK1/Akt/mTOR signaling inhibitor	*Ipomoea batatas* polysaccharides (IBPs)	Lung cancer	[107]
	PI3K inhibitor	PF-04691502	Solid tumor	[108]
	mTOR inhibitor	Everolimus	Acute lymphoblastic leukemia	[109]
	mTOR inhibitor	Ridaforolimus (Deforolimus)	Soft-tissue sarcoma	[110]
	mTOR inhibitor	Temsirolimus	Renal cell carcinoma	[111]
	mTOR inhibitor	Sorafenib	Renal cancer, hepatocellular cancer	[112]
	mTOR inhibitor	Resveratrol	Ovarian cancer	[113,114]
	mTOR inhibitor	AZD-8055	Advanced solid tumor	[115]
	mTOR inhibitor	Rapamycin	Breast cancer, bladder cancer	[116]
	mTOR inhibitor	Torin 2	Hepatocarcinoma	[117]
	mTOR inhibitor	AZD8055	Acute myeloid leukemia	[118]
	mTOR inhibitor	WYE-354	Colon cancer	[119]
	MEK1/2 inhibitor	Selumetinib (AZD6244)	Colorectal cancer	[120]
Nucleation	Bcl-2 inhibitor	Obatoclax	Head and neck squamous cell carcinoma	[121]
	Bcl-2 inhibitor	Quercetin	Ovarian cancer	[122]
	BH3 mimetic	Gossypol	Hepatocellular carcinoma	[123]
Initiation/ Nucleation	TKI inhibitor	Erlotinib	Non-small cell lung cancer	[124]
	TKI inhibitor	Sunitinib	Clear cell ovarian carcinoma	[125]
	Microtubule-stabilizing agents	Docetaxel	Prostate cancer	[126]
	Phosphatidylinositol transfer protein alpha/beta	Microcolin H	Gastric cancer	[127]
	mAB against the folate receptor alpha	MORAB-003	Ovarian cancer	[128]
	Antiparkinsonian drug	Metixene	Metastatic brain cancer	[129]
	Alkylating agent	Temozolomide	Glioblastoma multiforme	[130]
	Disruption of androgen receptor signaling	Sulforaphane	Prostate cancer	[131]
	Proteasome inhibitor	Bortezomib	Multiple myeloma	[92]
		**Inhibitor**		
Initiation	ULK1 inhibitor	SBI-0206965	Neuroblastoma, non-small cell lung cancer	[132,133]
	ULK1/2 inhibitor	DCC3116	Lung cancer	[134]
Nucleation	PIK3C3/Vps34 inhibitor	SAR405	Renal cell carcinoma	[135]
	Vps34 inhibitor	VPS34-IN1	Acute myeloid leukemia	[136]
	Dual inhibitor of Type I and III PI3K inhibitor	Wortmannin	Lung cancer, breast cancer	[137]
Elongation	Atg4 inhibitor	Tioconazole	Colorectal cancer, breast cancer, glioma	[138]
	Atg4 inhibitor	S130	Colorectal cancer	[139]
	Atg4 inhibitor	FMK-9a	-	[140]
Degradation	v-ATPase inhibitor	Bafilomycin A1	Breast cancer	[141]
	Phagosome–lysosome fusion inhibitor	Monensin	Lung cancer	[142]
	Lysosome inhibitor	Hydroxychloroquine	Prostate cancer, melanoma	[143]
	Lysosomal autophagy inhibitor	Lys05	Thyroid cancer	[144]
	Lysosomal autophagy inhibitor	ROC-325	Renal cell carcinoma	[145]
	Multi-step inhibitor of autophagy	Verteporfin	Osteosarcoma	[146]
	USP10 and 13 inhibitor	Spautin-1	Chronic myeloid leukemia	[147]
	NEDD8 inhibitor	Pevonedistat(MLN4924)	Mantle cell lymphoma	[148]
	Protein-palmitoyl thioesterase 1 (PPT1) inhibitor	DQ661	Melanoma	[149]

Several studies have reported the role of autophagy in increasing drug resistance in tumor cells [150,151,152]. Autophagy can induce drug resistance to protect cells, posing significant clinical challenges to achieving successful cancer treatment [153]. Resistance to anticancer drugs can be overcome by the pharmacological inhibition of key components of the autophagy pathway [154]. Our laboratory has also reported that autophagy deficiency through *ATG5* knockout can increase sensitivity to the anticancer drugs paclitaxel and gossypol [155,156]. Therefore, cotreatment with autophagy inhibitors and other conventional cancer therapies could aid in developing new cancer treatment strategies to overcome drug resistance [157].

## 6. Challenges in Developing Anticancer Drugs Targeting Autophagy

The effect of autophagy on tumors is complex and multifaceted [158,159,160]. Chronic pan-autophagy inhibition can lead to significant limitations, including toxicity to multiple organs and potential tumor formation in mouse models where key autophagy genes are inactivated [161]. Conversely, the overactivation of autophagy pathways can make certain cell types more susceptible to cell death [162]. Therefore, regulation of autophagy is a critical and complex therapeutic strategy for improving cancer treatment [163,164,165].

However, the role of alternative autophagy in tumor development has not been fully elucidated. Our laboratory has reported that under unfavorable survival conditions, mitochondrial clearance through alternative autophagy rather than canonical autophagy could be a new survival strategy for tumor cells [20]. We found that Ras-transformed cells maintained their survival by downregulating canonical autophagy at high cell confluency. This suggests that, in such cases, autophagy inhibitors may be less effective in achieving anticancer effects. Therefore, strategies to regulate alternative autophagy should be considered. Canonical autophagy and alternative autophagy proceed through different mechanisms in the initial stages but follow similar processes when forming the autolysosome. Therefore, drugs that inhibit the fusion of autophagosomes and lysosomes could potentially inhibit not only canonical autophagy, but also alternative autophagy.

**Figure 3:**
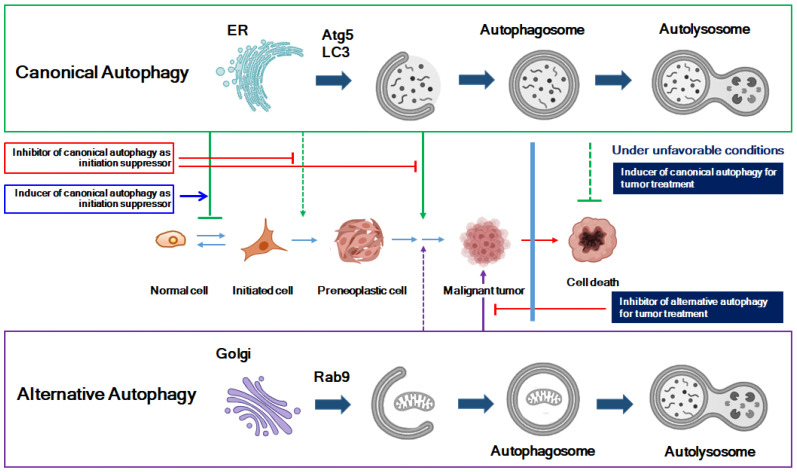
Anticancer drug development strategy based on the changing role of autophagy in tumorigenesis stages. Canonical ATG5/LC3-dependent autophagy suppresses tumor formation in the early stages of tumorigenesis but plays a crucial role in cell survival during the advanced stages of cancer. Therefore, for effective tumor therapy, it is important to use inducers of canonical autophagy during the initiation stage of tumor formation and inhibitors during the progression stage. Our research indicates that in challenging microenvironments for tumor cell survival, such as high cell confluency and exposure to anticancer drugs, alternative autophagy is more critical for maintaining cell survival than canonical autophagy. Thus, from a therapeutic perspective for advanced tumors, it is vital to develop anticancer strategies that focus on using inhibitors of alternative autophagy rather than canonical autophagy, depending on the tumor microenvironment.

Figure 3 shows a proposed model for a potential anticancer drug development strategy that considers the roles of canonical and alternative autophagy in tumorigenesis. Canonical autophagy suppresses tumor formation in the early stages of tumorigenesis but plays an important role in cell survival in the advanced stages of cancer. Therefore, it is important to use canonical autophagy inducers at the initiation stage of tumorigenesis and inhibitors at the progression stage for effective treatment. In addition, our study showed that alternative autophagy is more important than canonical autophagy in maintaining cell survival in a microenvironment that is difficult for tumor cell survival. Thus, it is important to develop an anticancer strategy that focuses on using inhibitors for alternative autophagy rather than canonical autophagy depending on the tumor microenvironment as a treatment for advanced tumors.

Another major unresolved issue in the development of drugs targeting autophagy is the lack of robust autophagy assays and biomarkers [29]. The LC3 protein, the most widely used marker in autophagy measurements, is increasingly reported to be associated with novel pathways, such as LC3-associated phagocytosis and LC3-associated endocytosis [166,167]. More importantly, LC3 accumulation cannot distinguish between the induction of autophagic flux and suppression of lysosomal activity. Therefore, current overreliance on LC3 may lead to errors in the measurement of canonical autophagy. The issue of measuring autophagic processes can confuse whether a drug developed to target autophagy acts as an inducer or an inhibitor. Moreover, it was recently discovered that LC3 lipidation does not occur during alternative autophagy [12]. Rab9 and Syntaxin 7 appear to be more appropriate potential markers [19]. To date, the most appealing method for quantifying alternative autophagy is to measure autolysosomes, which are dually stained with antibodies against the autophagosome marker Rab9 and the lysosome marker Lamp2, using fluorescence microscopy or flow cytometry.

## 7. Conclusions

The role of autophagy in cancer is complex, and our current knowledge in this field is very limited. To date, the two main roles of autophagy identified in tumorigenesis stages are as follows: basal autophagy generally suppresses tumor formation by inhibiting DNA damage and genomic instability in the early stages of tumor development, and conversely, autophagy promotes tumor progression in most cancers. Therefore, it is essential to investigate whether protective and cytotoxic autophagy can be simultaneously induced within cells, and how cells balance and regulate these processes. Based on the dual role of autophagy, cancer treatment strategies that combine autophagy inhibitors and inducers have been proposed [168]. In particular, inhibition of the late stages of autophagy suggests that the cytotoxic effects of autophagy inducers may be enhanced, contributing to tumor chemotherapy [169]. Therefore, a better understanding of the role of autophagy at different stages of tumor development is crucial for developing novel and effective therapeutic strategies for cancer treatment.

## Data Availability

This is a review and the majority of the article’s references are cited appropriately in the manuscript.
